# Enlarged Egg Size Increases Offspring Fitness of a Frog Species on the Zhoushan Archipelago of China

**DOI:** 10.1038/s41598-019-48147-8

**Published:** 2019-08-12

**Authors:** Feng Xu, Weikang Yang, Yiming Li

**Affiliations:** 1 CAS Key Laboratory of Biogeography and Bioresource in Arid Land, Xinjiang Institute of Ecology and Geography, Chinese Academy of Sciences, 818 South Beijing Road, Urumqi, Xinjiang, 830011 China; 2 Key Laboratory of Animal Ecology and Conservation Biology, Institute of Zoology, Chinese Academy of Sciences, 1 Beichen West Road, Chaoyang, Beijing, 100101 China; 3 Mori Wildlife Ecological Monitoring and Experimentation Station, Xinjiang Institute of Ecology and Geography, Chinese Academy of Sciences, 818 South Beijing Road, Urumqi, Xinjiang, 830011 China; 4 University of Chinese Academy of Sciences, Beijing, 100049 China

**Keywords:** Herpetology, Biogeography

## Abstract

Egg size represents maternal investment and is an important life-history trait. It also is assumed to have an effect on offspring fitness. Life-history theory predicts that oviparous animals on islands will produce enlarged eggs because of increased maternal investment to improve offspring performance to facilitate intra-specific competition. The life-history theory, developed during the 1950s, provides a possible explanation for the “island rule”, but this rule has seldom been tested. Although several studies have detected a positive relationship between egg size and offspring fitness, it is difficult to exclude the covarying effects on offspring performance, such as genetic variation and developmental plasticity; predictions made using the life-history theory on the islands have not been tested. In this study, we have evaluated the relationship between egg size and offspring fitness on 20 islands in the Zhoushan Archipelago and two nearby mainland sites. To exclude covarying effects, we compared larval performance among different egg sizes in three levels: among siblings within clutches, among clutches within populations, and among different islands. The results showed that frogs on most of the islands did produce enlarged eggs and that their larvae had improved larval fitness. Additionally, at all three levels, the offspring that evolved from enlarged eggs had increased offspring fitness. The results of this study indicate that, for the first time, the life-history theory predictions concerning egg size and offspring fitness are supported.

## Introduction

Egg size is an important life-history trait that can affect offspring fitness, such as size at hatching, survival, growth rates, and stress tolerance, in many animals, including insects, amphibians, reptiles, and birds^1–5^.

Variation in egg size is widespread in oviparous animals. The maternal investment, genetic variation, and developmental plasticity were proposed as the causes of this variation, and were also thought to have an impact on offspring fitness^6–9^. Among these factors, maternal investment is of particular interest for evolutionary ecologists; however, whether maternal investment alone is qualified to cause the egg size variation and influence offspring fitness has seldom been investigated^9,10^.

Ever since Darwin studied evolution, his theories have played an important role in evolutionary biology and ecological studies^11^. Animals on islands often show distinctive differences in life-history traits, such as body size and egg size, when compared with neighboring mainland populations; this is known as the island rule^12–15^. Until recently, there has not been a good explanation for the island rule, except for the life-history theory^12^; which proposes that animals on an island invest more energy in maternal investment to each offspring compared with the nearby mainland, and this increased investment provides their offspring with a higher level of fitness to meet the needs of greater intra-specific competition^12,13,16^. Although the life-history theory provides an ideal explanation for the trait differences on the island, the evolutionary consequences of these changes on offspring fitness has seldom been tested^12–14^. For oviparous species with no parent care, such as the rice frog (*Fejervarya limnocharis*), the egg is the only way for an adult to invest energy in her offspring, and the maternal investment can be estimated accurately by egg size^17^. Therefore, the rice frogs present on the islands provide an ideal chance to investigate the effect of maternal investment on egg size variation and offspring fitness.

Although several studies have shown that egg size has a great impact on offspring fitness, large eggs do not always equate to high fitness. For example, some studies did not detect an advantage for tadpoles developing from large eggs^10,18–21^. The fitness of different sized eggs has been related to environmental conditions. For insects, such as the *Stator limbatus*, the advantage of large eggs was observed only under adverse conditions, such as starvation or desiccation^5,22^. Additionally, in one type of snake, *Tropidonophis mairii*, larval fitness was not related to egg size but was positively related with water uptake and clutch size^23,24^. In an Italian frog, *Rana latastei*, fitness was positively related with egg size when compared between different populations and clutches, but there was no correlation when compared among siblings within clutches^10^. Therefore, whether large eggs indicate increased maternal investment and whether these large eggs lead to increased fitness still need to be investigated in other species.

The rice frog is a small grey anuran with a wide distribution in China^25^, and it is one of the most abundant frog species in the study area^17,26^. They start breeding in late April and continue to late August. The breeding season lasts for approximately four months^17^. The rice frog has no parental care. Females lay 120 to 280 eggs each time^17^. Their eggs and tadpoles can be easily found in lentic water, such as rice fields, marshes, ditches, and small pools. The eggs can hatch within 48 hours, with the time depending on the temperature^17^. With easily found clutches and a short hatching time, rice frogs provide an ideal opportunity to test the effects of egg size on offspring fitness. In this paper, according to the life-history theory and the characteristics of rice frogs, we tried to answer the following questions: (1) does the rice frog on the island have higher maternal investment and offspring fitness; and (2) does the enlarged egg size cause high larval fitness for this species on the islands?

## Results

### Comparisons of egg size, hatching success, and tadpole size of the rice frog between the islands and the mainland

The egg size, tadpole size, and hatching success of the rice frog differed among the 20 island populations and the mainland population (One-way ANOVA, *F* = 13.737, *df* = 19, *p* < 0.001 for egg size; *F* = 24.871, *p* < 0.001 for tadpole size; *F* = 10.713, *p* < 0.001 for hatching success). The LSD multiple comparisons showed that for 17 out of 20 island populations of rice frog, egg size, hatching success, and tadpole size were significantly larger than nearby mainland populations. The populations of rice frogs on the three larger islands (Zhoushan, Daishan, Liuheng) did not differ with the mainland population (Tables 1 and 2).

**Table 1:** Life-history traits of the rice frog on 20 islands of the Zhoushan Archipelago and two mainland sites in China.

Site	Egg size Mean ± SE	P (t-test) df = 20	Tadpole size Mean ± SE	P (t-test) df = 20	Hatching success Mean ± SE	P (t-test) df = 20
Mainland	1.51 ± 0.04		4.82 ± 0.04		74.29 ± 1.81	
Zhoushan	1.51 ± 0.03	0.785	4.81 ± 0.03	0.872	77.57 ± 1.87	0.248
Daishan	1.52 ± 0.04	0.430	4.75 ± 0.05	0.221	79.09 ± 2.68	0.272
Liuheng	1.52 ± 0.03	0.393	4.88 ± 0.04	0.284	80.00 ± 1.97	0.243
Jintang	1.54 ± 0.02	**0.004**	4.93 ± 0.03	**0.044**	82.00 ± 3.00	**0.04**
Qiushan	1.55 ± 0.02	**<0.001**	4.94 ± 0.04	**0.021**	82.92 ± 3.17	**0.015**
Taohua	1.54 ± 0.03	**0.002**	4.96 ± 0.05	**0.01**	85.42 ± 3.11	**<0.001**
Dachangtu	1.55 ± 0.02	**<0.001**	4.97 ± 0.03	**0.006**	85.00 ± 2.34	**0.005**
Daxie	1.55 ± 0.02	**<0.001**	5.04 ± 0.04	**<0.001**	85.50 ± 2.29	**0.005**
Xiushan	1.56 ± 0.04	**0.005**	5.05 ± 0.07	**<0.001**	86.50 ± 3.50	**<0.001**
Sijiao	1.56 ± 0.02	**<0.001**	5.10 ± 0.04	**<0.001**	84.17 ± 2.88	**0.003**
Meishan	1.54 ± 0.03	**0.021**	5.15 ± 0.04	**<0.001**	89.55 ± 1.25	**<0.001**
Xaizhi	1.56 ± 0.04	**0.001**	5.24 ± 0.05	**<0.001**	91.36 ± 2.44	**<0.001**
Cezi	1.56 ± 0.02	**<0.001**	5.19 ± 0.02	**<0.001**	92.73 ± 2.17	**<0.001**
Denbu	1.58 ± 0.03	**<0.001**	5.25 ± 0.03	**<0.001**	93.00 ± 1.53	**<0.001**
Xiaochangtu	1.57 ± 0.02	**<0.001**	5.24 ± 0.06	**<0.001**	95.00 ± 2.24	**<0.001**
Changbai	1.58 ± 0.01	**<0.001**	5.33 ± 0.04	**<0.001**	94.09 ± 2.11	**<0.001**
Fodu	1.58 ± 0.03	**<0.001**	5.34 ± 0.05	**<0.001**	97.95 ± 0.63	**<0.001**
Dayushan	1.58 ± 0.02	**<0.001**	5.37 ± 0.04	**<0.001**	97.00 ± 1.11	**<0.001**
Damao	1.61 ± 0.03	**<0.001**	5.52 ± 0.05	**<0.001**	98.00 ± 1.11	**<0.001**
Huni	1.61 ± 0.02	**<0.001**	5.45 ± 0.03	**<0.001**	97.73 ± 1.04	**<0.001**

The LSD test was used for multiple comparisons. Estimates of egg size, tadpole size, hatching success of the Rice frog on the mainland and 20 islands of the Zhoushan Archipelago (significant results in bold type) are shown.

**Table 2:** Results of the generalized linear mixed model with tadpole size and hatching success of the rice frog to egg size compared among siblings within the clutch and among clutches within the population.

Offspring fitness	Explanatory variables	*ß*	SE	*P*
Tadpole size	Egg size (among siblings within clutch)	6293.68	200.42	<0.001
Tadpole hatching success	Egg size (among siblings within clutch)	24.93	1.81	<0.001
Tadpole size	Egg size (among clutches within population)	0.750	0.108	<0.001
Tadpole hatching success	Egg size (among clutches within population)	45.198	74.919	0.271

### Relationship between island characteristics and egg size, tadpole size, and hatching success of the rice frog

**Figure 1:**
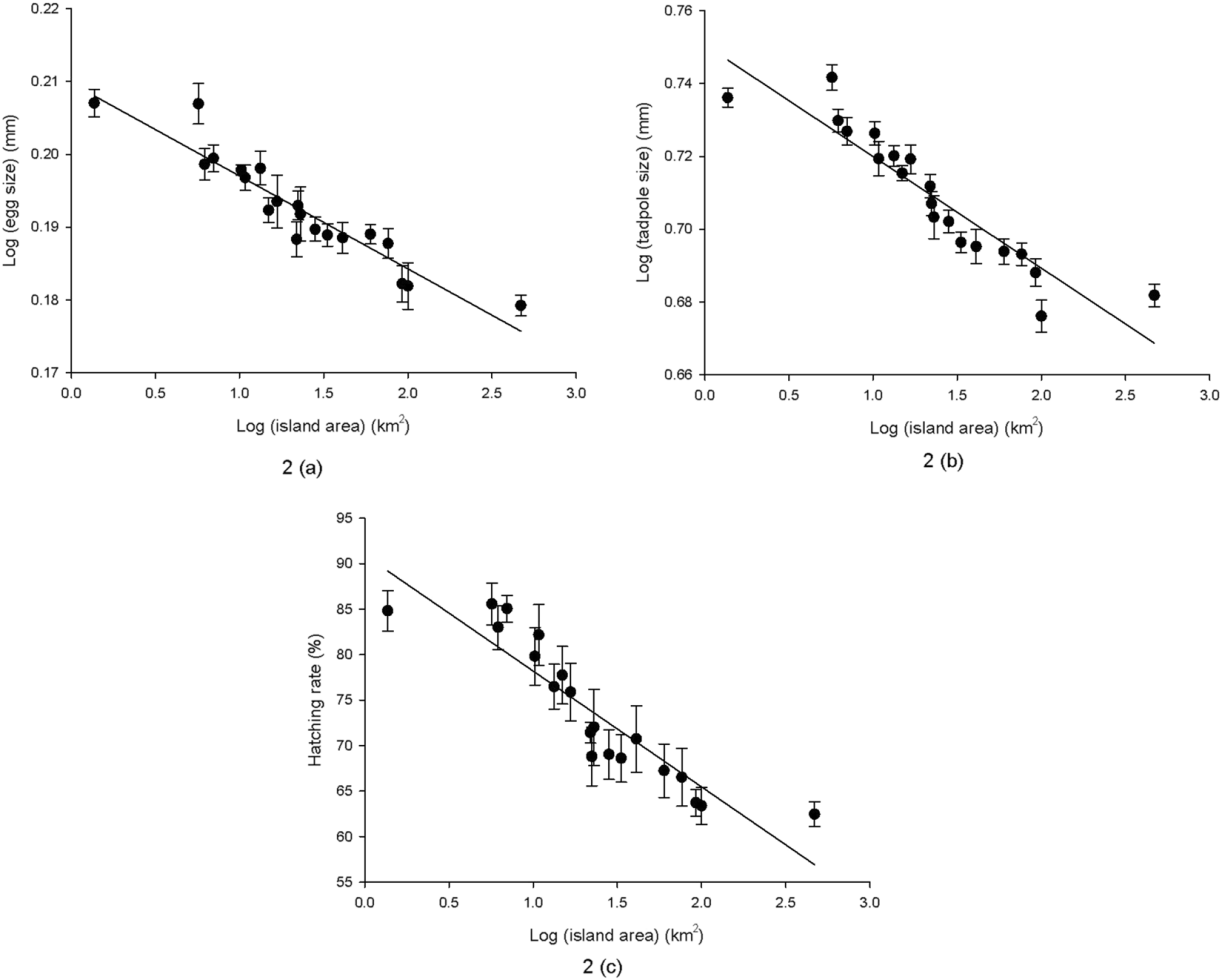
Relationships between island area and life-history traits of the rice frog in the Zhoushan Archipelago. (**2a**): egg size (*r* = −0.939, *p* < 0.001); (**2b**) tadpole size (*r* = 0.927, *p* < 0.001); (**2c**) hatching success (*r* = 0.933, *p* < 0.001).

Egg size, hatching success, and tadpole size were not related to an island’s distance to the mainland (*r* = −0.118, n = 20, *p* = 0.621 for egg size; *r* = −0.010, *p* = 0.968 for tadpole size; *r* = −0.035, *p* = 0.883 for hatching success), but all factors were significantly related to the island’s size (Fig. 1).

### Relationships between egg size and larval fitness

**Figure 2:**
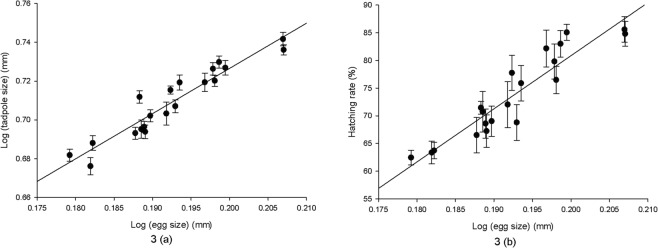
Relationships between egg size and offspring fitness of the rice frog in the Zhoushan Archipelago. (**3a**) egg size and tadpole size (*r* = 0.952, n = 20, *p* < 0.001); (**3b**) egg size and hatching success (*r* = 0.921, *p* < 0.001).

We compared relationships between egg size and offspring fitness at three levels: among siblings within the clutches, among clutches within populations, and among island populations. The generalized linear mixed model showed that egg size was significantly related to tadpole size and hatching success among siblings within clutches using the clutch and island as nested factors (Table 1). The model also showed that egg size was significantly related to tadpole size among clutches within populations using the island as a nested factor (Table 1). We used a partial correlation to detect the relationship between egg size and larval fitness among different island populations controlling the covarying effects of female body size, and the results showed that egg size was significantly related with tadpole size (*r* = 0.732, *n* = 20, *p* < 0.001) and hatching success (*r* = 0.533, *n* = 20, *p* = 0.019) (Fig. 2).

## Discussion

The rice frog produced enlarged eggs on the Zhoushan Archipelago, which negatively correlated with an island’s area. These results are consistent with previous studies^12,27^. According to the life-history theory, animals on islands encounter higher intra-specific competition than those on the mainland; therefore, they allocate more energy in maternal investment to each offspring, and this, in turn, results in offspring with higher fitness^12,13,16,27^. Our results supported this hypothesis: the rice frog did allocate more energy to maternal investment to produce larger eggs on the islands of the Zhoushan Archipelago. The results also indicated that island characteristics, represented by island size and inherent isolation, had an effect on the rice frog’s maternal investment: stronger on small islands than on big islands and the mainland.

The results indicated that rice frog offspring on the islands had a higher level of fitness than those on the mainland. Both the larval size and hatching success of the rice frog was larger on the islands, and were negatively related to the island’s area. These results are consistent with the life-history theory predictions of species’ development in island habitats^12,13,27^, which states that animals on islands encounter higher intra-specific competition; therefore, these animals need to allocate additional energy toward growth rather than reproduction because a large body size is more competitive than a smaller one^16,28^. At the same time, animals will invest more energy into each offspring to enlarge their fitness for future competition^12,13^. Therefore, for the island population, we found enlarged egg sizes, higher hatching successs, and larger larval sizes compared with nearby mainland populations^12,13,27^. Other studies also found increased offspring fitness for populations on the islands^29,30^, but their research contained a limited number of islands and did not exclude covarying effects, such as genetic variation and environmental influences on offspring fitness. In this research, we surveyed 20 islands and compared the fitness of rice frog offspring on islands of different sizes, which provides strong evidence of improved offspring fitness on the islands compared to the mainland.

Our research also shows a significant positive relationship between egg size and offspring fitness when compared among siblings within clutches, among clutches, and among different island populations. These results provided strong evidence that egg size based on maternal investment influenced egg fitness in terms of hatching success and tadpole size, excluding the effects of other factors, such as genetic variation and developmental plasticity^31^. Others have also found similar results between egg size and egg fitness when comparing different populations, but their results did not exclude the covarying factors^10,31^. Krist and Remes (2004) proposed that analyzing the relationship between egg size and offspring performance among siblings could exclude other covariation by comparing among siblings. By evaluating the relationship between egg size and offspring fitness based on our three levels–among siblings within clutches, among clutches within the population, and among different island populations–to exclude covarying factors, our results showed that maternal investment is the most supportive factor influencing offspring fitness. Ficetola and Bernardi (2009) analyzed the relationship between egg size and offspring fitness using similar methods, and their results indicated that the advantages of enlarged egg size were not significant when compared among siblings within clutches, but they were significant when compared among clutches and among different populations. Therefore, they suggested that covariation between egg size and other effects may likely have influenced the final results in that study^10^. In our study, the results of the three levels were similar and indicated that egg size has an effect on offspring fitness; therefore, the egg size, viewed as the maternal investment, was possibly the important effect that influenced offspring fitness in this study. So, our results provide strong evidence for the life-history theory explanation of the island rule associated with egg size effects on offspring fitness^12,13^.

In summary, we found a significant increase in maternal investment and higher levels of offspring fitness in the rice frog on the 20 islands of the Zhoushan Archipelago compared to mainland populations; and the differences among islands were significantly related to the land mass of the island areas. By performing comparisons among siblings within clutches, among clutches, and among different island populations, we found significant positive relationships between egg size and offspring fitness. The results showed that egg size as an indication of maternal investment affects offspring fitness, thereby providing strong evidence for the life-history theory explanation of the island rule.

## Materials and Methods

### Study area

**Figure 3:**
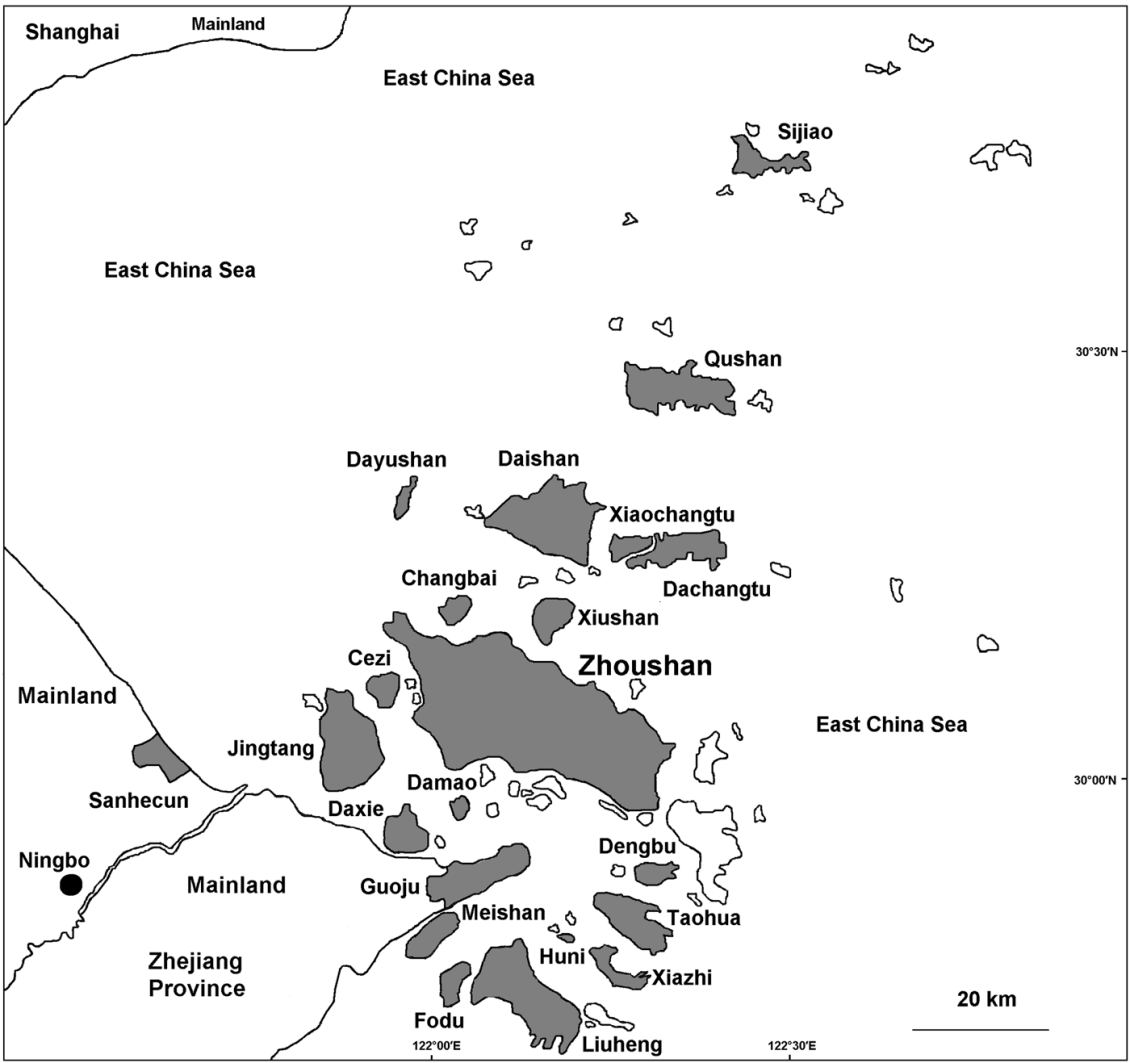
Map of sample sites on the Zhoushan Archipelago and on the nearby mainland in Eastern China. Grey color represents the sampled sites on 20 islands in the archipelago and the mainland.

The Zhoushan Archipelago (29°31′–31°04′N, 121°30′–123°25′E) is the largest archipelago in China, comprising 1339 islands in the East China Sea (Fig. 3). The archipelago was originally a part of the neighboring mainland, but it separated approximately 7000–9000 years ago. The islands and nearby mainland area, however, still share similar flora and fauna^32^. Both the mainland and archipelago are covered with subtropical evergreen, broad-leafed forest and experience highly seasonal weather conditions, with a mean temperature ranging from 5.7 °C in January to 26.7 °C in July. There are numerous suitable habitats and breeding sites for the frogs on the island, such as freshwater ponds, pools, ditches, and rice fields. Species richness is poorer on the islands than on the mainland, with 10 amphibian species found in the Zhoushan Archipelago compared with 17 species on the nearby mainland; no endemic vertebrate species are found on the islands^17,26,33,34^.

### Data collection

The study was conducted from early June to late August during the rice frog breeding season in 2009 on two mainland sites (Guoju and Sanhecun) and 20 islands (Zhoushan, Daishan, Liuheng, Jingtang, Qushan, Taohua, Dachangtu, Daxie, Meishan, Xiushan, Sijiao, Xiazhi, Denbu, Cezi, Changbai, Xiaochangtu, Fodu, Dayushan, Damao, and Huni) (Fig. 3). At each site, we collected data on egg size, tadpole size, hatching success, and female body size. Among these sampled sites, Zhoushan Island was surveyed three times at the beginning, middle, and end of the breeding season to compare life-history trait variations within a season and to minimize the potential effects of sampling time. The survey sequence of the 20 islands was randomly chosen with the help of a random number table. The survey was carried out for four to six days at each location^27,35^.

We searched for rice frog clutches every morning between 5:30 and 9:30 am. Once found, the entire clutch was collected and placed in a plastic bag for further analysis in the laboratory. Rice frogs were captured by hand. Females were placed in black plastic bags or net bags with holes for airflow and taken back to the laboratory for measurement. The snout-vent length (SVL) was measured with Vernier calipers to the nearest 0.02 mm^27^. We used SVL to represent body size because body weight changes dramatically for adult female frogs depending on physiological states, such as bladder fullness and egg formation^27,36,37^. We identified the gender of sexually mature rice frogs according to the presence or absence of secondary sexual characters, such as nuptial pads and black pigment on the throat^17,25,35^. Eggs and frogs were released the following morning at the capture site.

The clutch was spread out in a white tray for counting clutch size. We then randomly chose 20 eggs from each clutch to measure their diameter to the nearest 0.02 mm using Vernier calipers, and with the help of a magnifying glass, we calculated the average egg size for each clutch^38,39^. Because the embryo changes to an oval shape after the Gosner 10 stage, only eggs prior to this developmental stage were measured^40^. After measuring, each egg was placed in a plastic cubic cell (10 × 10 × 10 mm) filled with aged tap water. Each cubic cell panel contained 100 cells. The panel was placed in a constant-temperature incubator for hatching at 28 °C, which is the same temperature as the mean outdoor water temperature in August^17^. Offspring fitness was calculated by hatching success and tadpole length^41^. After 48 hours, we checked for the hatching success and measured hatchling tadpole size. Tadpole size was measured from digital images using the digital image analysis software ImageJ^41^.

Data on island areas were obtained from Chen (1989), and the distances of each island to the mainland and to the nearest big island were measured on a map with a scale of 1:400,000^26,34,35,42^.

### Statistical analysis

The data on egg size, tadpole size, female body size and island characteristics were log-transformed to meet the assumptions of normality. Hatching success was arcsine square-root transformed for normality^43^.

A preliminary analysis showed that the life-history traits did not differ significantly between the two mainland sites (Guoju and Sanhe) (*t-*test, *t* = 0.285, *df* = 33, *p* = 0.780 for egg size; *t* = 1.488, *p* = 0.146 for tadpole size; and *t* = 0.506, *p* = 0.616 for hatching success); and between the three surveys within a breeding season on Zhoushan Island (One-way ANOVA, *F* = 0.505, *df* = 2, *p* = 0.608 for egg size; *F* = 0.642, *p* = 0.533 for tadpole size; *F* = 0.020, *p* = 0.980 for hatching success). Therefore, we pooled the data from these respective groups as a new dataset to increase the sample size^27^. We used one-way ANOVA and LSD multiple comparison to examine differences in life-history traits between the mainland populations and each island population. We used simple linear regression to examine the relationships between island characteristics and life-history traits^43^.

The difficulty in identifying the effect of egg size on offspring fitness is to exclude the covarying effects of genetics and non-genetic factors. Krist and Remes (2004) proposed analyzing the relationship between egg size and performance among siblings or experimentally manipulating egg size to evaluate the effect of egg size independently from genetic or other maternal effects. We followed their method to compare the fitness of large and small eggs at three levels: among siblings within clutches, among clutches within the island, and among different islands^31^. When looking within clutches and within islands, offspring fitness was compared using generalized linear mixed models of survival rate and larval size, where the clutch effect and island effect were included as nested effects^10^. We used partial regression to detect the relationship between egg size and offspring fitness among different island populations and controlled female SVL effects to exclude their covarying effects on egg size and offspring fitness.

The one-way ANOVA, LSD multiple comparisons, simple linear regressions, and partial regressions were performed using SPSS (SPSS Inc., 1998). The generalized linear mixed model was performed in R 2.8.1^44^. All tests were two-tailed and statistical significance was set at P ≦ 0.05.

## Data Availability

The datasets generated and/or analyzed during the current study are available from the corresponding author on reasonable request.
